# 以Löeffler心内膜炎为主要临床特征的嗜酸性粒细胞增多症6例临床分析

**DOI:** 10.3760/cma.j.cn121090-20250106-00008

**Published:** 2025-09

**Authors:** 亚男 马, 苏宁 陈, 炳元 周, 小飞 杨

**Affiliations:** 1 苏州大学附属第一医院血液科，江苏省血液研究所，国家血液系统疾病临床医学研究中心，国家卫生健康委员会血栓与止血重点实验室，苏州 215006 National Clinical Research Center for Hematologic Diseases, Key Laboratory of Thrombosis and Hemostasis of Ministry of Health, Jiangsu Institute of Hematology, the First Affiliated Hospital of Soochow University, Suzhou 215006, China; 2 南通大学第四附属医院、盐城市第一人民医院血液科，盐城 224000 Department of Hematology, the Fourth Affiliated Hospital of Nantong University, Yancheng First People's Hospital, Yancheng 224000, China; 3 苏州大学附属第一医院心血管内科，苏州 215006 Department of Cardiology, The First Affiliated Hospital of Soochow University, Suzhou 215006, China

## Abstract

为提高对Löeffler心内膜炎的认识，回顾性分析2019年1月至2024年10月期间苏州大学附属第一医院收治的6例以Löeffler心内膜炎为主要临床特征的嗜酸性粒细胞增多症患者。6例患者中男性5例，中位年龄45.5（31～77）岁，所有患者外周血WBC、嗜酸性粒细胞计数均有增高，临床症状及影像学检查考虑为Löeffler心内膜炎，2例合并脑梗死。5例患者完善了融合基因检测，例4、例5检测到FIP1L1::PDGFRA阳性，例6诊断为TLS::ERG（+）急性髓系白血病。2例FIP1L1::PDGFRA阳性患者经伊马替尼治疗后迅速缓解；例6经糖皮质激素、羟基脲治疗后症状缓解，后接受IAG（阿糖胞苷+伊达比星+G-CSF）方案化疗后完全缓解，并于8个月后进行异基因造血干细胞移植，移植后1年因本病复发合并感染死亡。其余3例患者经糖皮质激素治疗后好转。除例6外其余患者至随访终点均存活，例4停用伊马替尼后28个月复发，再次启动治疗后缓解。

## Abstract (en)

To improve the understanding of Löeffler endocarditis, we retrospectively analyzed six patients with eosinophilia, predominantly characterized by Löeffler endocarditis, who were treated at the First Affiliated Hospital of Soochow University from January 2019 to October 2024. Among the 6 patients, 5 were male, with a median age of 45.5 years（31–77 years）. All patients demonstrated increased white blood cell count and eosinophil count in peripheral blood. Clinical symptoms and imaging examination were considered as Löeffler endocarditis, and two cases were complicated by cerebral infarction. Fusion gene testing was completed in five patients, with cases 4 and 5 demonstrating FIP1L1::PDGFRA fusion gene positivity, and case 6 with TLS::ERG（+）acute myeloid leukemia. Two patients with FIP1L1::PDGFRA positivity achieved rapid remission after imatinib treatment. Case 6 experienced symptom relief after glucocorticoids and hydroxyurea treatment, followed by complete remission after chemotherapy with the cytarabine（20 mg q12 h × 14 days）, idarubicin（5 mg on days 2–12 every other day）, and G-CSF（adjusted based on blood counts）regimen. After 8 months, the patient underwent allogeneic hematopoietic stem cell transplantation but died 1 year post-transplantation due to disease relapse complicated by infection. The remaining three patients demonstrated improvement after glucocorticoid treatment. Except for case 6, the other patients were still alive, with case 4 relapsing at 28 months after imatinib discontinuation and relieved after treatment re-initiation.

嗜酸性粒细胞增多症（Eosinophilia）是一组异质性疾病，部分患者伴有器官损伤即高嗜酸性粒细胞综合征（Hypereosinophilic syndrome, HES）。Löeffler心内膜炎于1932年首次被报道[1]，是一种以外周血嗜酸性粒细胞增多和炎症性血栓形成为特征的罕见疾病，嗜酸性粒细胞侵袭心内膜、心肌引起心内膜坏死、血栓形成及心肌纤维化导致多种并发症，其病因主要取决于嗜酸性粒细胞增多的原因，包括继发性、克隆性、遗传性以及特发性因素[2]–[3]。本病较为罕见，临床表现无特异性，易出现漏诊误诊。为提高对Löeffler心内膜炎的认识，本研究回顾性分析我院诊治的6例以Löeffler心内膜炎为主要临床特征的嗜酸性粒细胞增多症患者的临床及实验室特征，并总结文献报道病例进行分析。

## 病例与方法

1. 病例：本研究纳入苏州大学附属第一医院2019年1月至2024年10月收治的6例以Löeffler心内膜炎为主要临床特征的嗜酸性粒细胞增多症患者。Löeffler心内膜炎诊断标准参照HES：①2次外周血检查嗜酸性粒细胞绝对计数>1.5×10^9^/L（间隔时间>1个月）；②伴有心脏损害的证据，如胸痛、胸闷、呼吸困难等临床表现等，心肌损伤标志物的升高、ST段抬高或T波倒置以及心内膜增厚或心尖部血栓等；③排除其他潜在的疾病作为心脏损伤的主要驱动因素[3]。心内膜活检是诊断金标准。

2. 资料采集：采集患者的临床资料及随诊记录，包括：①一般信息：性别、年龄、初诊时间、科室；②临床特点：主诉、症状、体征、基础疾病等信息；③实验室检查：血常规、生化指标、骨髓细胞形态学、白血病免疫分型、细胞遗传学、分子生物学等检查；④影像学检查：超声心动图、心脏磁共振（CMR）、胸腹部CT等；⑤治疗方案及预后情况，疗效评估。

3. 文献荟萃分析：以“Löeffler心内膜炎”“心内膜炎”“Löeffler endocarditis”为关键词在CNKI及Pubmed数据库进行检索，汇总2023–2024年相关文献，获得49例病例[4]–[49]，分析其临床特征。

4. 随访：采用电话、查阅患者住院病历及门诊病历的方式随访，随访截止日期为2024年12月31日，中位随访时间为38（2.5～69）个月，所有患者均未失访。

5. 统计学处理：使用GraphPad Prism 10软件进行统计分析，定量数据（不符合正态分布）以中位数（范围）表示，应用 *t*检验进行分析；定性资料采用卡方检验进行分析。*P*<0.05为差异有统计学意义。

## 结果

1. 临床特征：6例患者血常规均提示嗜酸性粒细胞增多，中位WBC 16.9（10.08～42.40）×10^9^/L、嗜酸性粒细胞计数9.55（2.19～25.27）×10^9^/L、HGB 133.5（90～151）g/L、PLT 89（45～239）×10^9^/L；完善超声心动图或CMR检查并考虑Löeffler心内膜炎，其中男5例，女1例，中位年龄45.5（31～77）岁。6例患者自身抗体、过敏源、IgE、寄生虫检测均为阴性。其中有5例患者完善了融合基因检测，2例（例4、例5）FIP1L1::PDGFRA融合基因阳性，例6 TLS::ERG融合基因阳性，并最终诊断为TLS::ERG（+）AML。6例患者均有胸闷气促表现，其中1例年轻男性合并脑梗死症状，表现为头晕、言语不清。头颅MRI检查有2例患者合并脑梗死，50％患者合并脾脏肿大。患者临床资料详见表1。

**表1:** 6例以Löeffler心内膜炎为主要临床特征的嗜酸性粒细胞增多症患者基本资料

例号	性别	年龄（岁）	主诉	WBC（×10^9^/L）	EO（×10^9^/L）	HGB（g/L）	PLT（×10^9^/L）	BNP（µg/L）	骨髓原始细胞（％）	骨髓EO（％）	染色体核型	融合基因（PCR/RNAseq）	NGS
1	女	77	胸闷气促	14.72	2.19	135	239	26 419	未做	未做	未做	未做	未做
2	男	33	头晕、胸闷、言语不清	23.39	8.96	151	115	3 500	未做	未做	未做	–	–
3	男	50	胸闷气促	10.08	3.24	145	68	1 021	0	26.5	正常核型	–	–
4	男	41	胸闷气促	18.76	11.01	120	82	7 725	0	27.0	正常核型	FIP1L1::PDGFRA	–
5	男	50	胸闷气促	15.04	10.14	132	96	1 920	0	15.0	正常核型	FIP1L1::PDGFRA	–
6	男	31	胸闷、发热	42.40	25.27	90	45	4 935	41.5	32.5	46,XY,t（16;21）（p11;q22）[10]	TLS::ERG	PTPN11

**Table:** 

例号	脾脏	超声心动图	CMR	头颅MRI	治疗方案
1	正常	左室腔内异常回声	未做	未做	糖皮质激素
2	脾大	左右室腔异常回声	左室腔中部以下及右室心尖部异常信号，左室心尖部舒张期室壁增厚，左右室壁乳头肌水平以下活动幅度减弱，左室壁心尖部见明显强化	两侧额顶枕叶多发亚急性梗死，脑内多发梗死灶	糖皮质激素
3	正常	左室腔内异常回声	左室腔容积减小；左室壁增厚，以近心尖部明显，伴室壁延迟强化；左室腔异常信号	双侧额顶叶、半卵圆中心多发亚急性期梗死可能	糖皮质激素
4	正常	左室心尖心内膜增厚伴闭塞	心内膜凹凸不平，双室心尖部室腔缩小	未做	伊马替尼
5	脾大	左心室多壁段心内膜增厚、回声增强伴室壁舒张活动减弱	左室心尖部为主心内膜下延迟强化及附壁血栓形成，伴局部心腔缩小	未做	伊马替尼
6	脾大	左室腔中部至心尖部新月形中等回声附着于心内膜，心尖部增厚，心尖部心腔明显缩小	未做	未做	IAG方案，8个月后行allo-HSCT

**注** EO：嗜酸性粒细胞；BNP：重组人脑利钠肽；PCR：逆转录聚合酶链反应；RNA-seq：转录组测序；NGS：二代测序；CMR：心脏磁共振；MRI：磁共振；IAG方案：阿糖胞苷（20 mg，每12 h 1次×14 d）+伊达比星（5 mg，第2～12天，隔日1次）+G-CSF（根据血常规调整）；allo-HSCT：异基因造血干细胞移植

2. 治疗方案及预后：4例患者初诊于心血管内科，例2初诊于神经内科，仅例6初诊于血液科。例1、2、3未检测到血液肿瘤相关融合基因，给予抗凝及糖皮质激素治疗后症状好转，嗜酸性粒细胞计数逐步下降至正常。例4、5初诊时予糖皮质激素治疗，嗜酸性粒细胞计数下降不理想，血液科会诊后完善骨髓细胞形态学、免疫学、细胞遗传学、分子生物学（MICM）检查后检出FIP1L1::PDGFRA融合基因，予伊马替尼治疗后迅速达到完全血液学缓解（CHR），例4于2021年7月19日（伊马替尼治疗后6个月）复查FIP1L1::PDGFRA融合基因转阴。例6初诊经糖皮质激素、羟基脲治疗后症状缓解，接受了IAG（阿糖胞苷20 mg，每12 h 1次×14 d；伊达比星5 mg，第2～12天，隔日1次；G-CSF，根据血常规调整）方案化疗后完全缓解，8个月后行allo-HSCT，移植后1年本病复发合并感染死亡。截至2024年12月31日，其余5例患者均为存活状态，例4于2022年6月自行停药，2024年10月8日来院复查血常规：WBC 11.59×10^9^/L、嗜酸性粒细胞计数6.22×10^9^/L、HGB 159 g/L、PLT 111×10^9^/L，RT-PCR检测FIP1L1::PDGFRA/ABL1 12.65％，再次启动伊马替尼治疗，1个月后复查血常规恢复正常。

3. 国内外文献报道的Löeffler心内膜炎病例临床特征：汇总国内外文献报道的Löeffler心内膜炎病例共计49例，男28例（56％），女21例（44％），中位年龄50（6～76）岁。44例患者有详细的嗜酸性粒细胞计数记录，中位嗜酸性粒细胞计数为8.945（0.53～53.98）×10^9^/L，其中3例患者为轻度嗜酸性粒细胞增多，其余均>1.5×10^9^/L。49例患者均完善超声心动图或CMR检查，14例进行了心内膜活检证实Löeffler心内膜炎诊断；多器官系统评估发现11例患者合并其他脏器浸润，其中5例合并脑梗死[4]–[49]。

4. 国内外文献报道不同病因所致Löeffler心内膜炎临床特征比较：48例患者明确了Löeffler心内膜炎病因，包括：2例急性淋巴细胞白血病（ALL）、1例AML、1例弥漫大B细胞淋巴瘤、6例FIP1L1::PDGFRA阳性的髓系和淋系肿瘤伴嗜酸性粒细胞增多症和酪氨酸激酶基因融合（myeloid/lymphoid neoplasms with eosinophilia and tyrosine kinase gene fusions，M/LN-eo-TK）、1例JAK2 V617F突变阳性的骨髓增殖性肿瘤（MPN）、2例JAK2少见位点突变的慢性嗜酸性粒细胞白血病（CEL）、4例药物反应、1例寄生虫病、1例类风湿性关节炎以及29例特发性因素。将血液肿瘤作为主要病因的病例与特发性病例进行比较，年龄分布差异无统计学意义，血液肿瘤组中男性比例更高，外周血嗜酸性粒细胞计数更高，差异具有统计学意义（*P*<0.01）（表2）。

**表2:** 国内外文献报道不同病因所致Löeffler心内膜炎临床特征比较

组别	例数	性别（例，男/女）	年龄［岁，*M*（范围）］	EO［×10^9^/L，*M*（范围）］
血液肿瘤组	13	9/4	45（6～70）	16.94（3.05～53.98）
特发性嗜酸性粒细胞增多组	29	13/16	55（8～76）	6.21（0.53～30.10）

*P*值		0.143	0.102	0.006

**注** EO：嗜酸性粒细胞计数

## 讨论

HES是一组嗜酸性粒细胞增多浸润脏器的罕见疾病，SEER数据库显示发病率约0.035/10万，男性多见，中位年龄52.5岁[50]，13％～22％的HES患者出现心脏累及[51]，包括Löeffler心内膜炎。Löeffler心内膜炎临床表现无特异性，常见的症状包括呼吸困难、消瘦、疲劳、进行性心力衰竭、血管栓塞、发热、心包炎、脾肿大等[3]，易出现漏诊误诊。心内膜活检是确诊Löeffler心内膜炎的“金标准”，可直接观察到心内膜纤维化和嗜酸性粒细胞浸润，但由于其创伤性及高风险受到限制[3]。超声心动图是评估HES心脏累及的常用手段，但是在Löeffler心内膜炎初期超声多表现为正常，后期可检测到心内膜增厚、心室内血栓及心室限制性充盈障碍[52]。与超声心动图相比，CMR具有更高的灵敏度和特异性，可以发现心脏受累的早期阶段（急性心肌炎/坏死阶段）的心肌异常，并通过钆对比剂增强显像，能够特异性识别心内膜下纤维化和炎症区域，与其他限制性心肌病区分[53]。因此，在临床工作中更多应用CMR或超声心动图，结合外周血嗜酸性粒细胞>1.5×10^9^/L及临床表现进行Löeffler心内膜炎诊断。

Löeffler心内膜炎病因复杂，特发性嗜酸性粒细胞增多是最为常见的病因[54]。我们总结了2023–2024年48例Löeffler心内膜炎病例发现大约60％为特发性因素所致，27％由血液肿瘤所致，其他因素包括自身免疫疾病、寄生虫感染、药物因素等。国内学者曾将特发性HES与PDGFRA/B重排的M/LN-eo-TK进行比较发现，PDGFRA/B重排的M/LN-eo-TK组男性多见且有更高的嗜酸性粒细胞计数（*P*<0.001）[55]。在Löeffler心内膜炎病例中，将特发性因素组与血液肿瘤组进行比较我们也得到了相似的结果。本中心的6例病例中有3例为恶性血液肿瘤，包括2例FIP1L1::PDGFRA（+）M/LN-eo-TK和1例TLS::ERG（+）AML。2003年Cools等[56]首次报道了FIP1L1::PDGFRA融合基因，该基因激活PDGFRA表达具有酪氨酸激酶活性的蛋白质，诱导嗜酸性粒细胞恶性增殖。关于FIP1L1::PDGFRA融合基因阳性病例的队列研究发现高达19％（28/151）的患者伴有心脏累及，其中17例表现为心肌内膜纤维化[57]。既往亦有多个FIP1L1::PDGFRA融合基因阳性Löeffler心内膜炎的个案报道[8]–[13]。因此对于Löeffler心内膜炎患者进行骨髓MICM检查非常必要。大多数FIP1L1::PDGFRA融合基因阳性患者对伊马替尼反应良好，并能达到完全分子学缓解（CMR），但43％的患者在停药后出现复发[57]。本研究中2例FIP1L1::PDGFRA融合基因阳性患者在伊马替尼治疗后迅速达到CHR，例4在停药28个月后出现复发，幸运的是再次启用伊马替尼治疗后依然迅速达到CHR，因此部分学者建议FIP1L1::PDGFRA融合基因阳性患者应该长期接受伊马替尼治疗[58]。

大约5％核心结合因子相关急性髓系白血病（CBF-AML）和1％ALL合并嗜酸性粒细胞增多[59]，由急性白血病（AL）诱导的Löffler心内膜炎更为罕见，近年来仅有少量个案报道，对于AL合并Löffler心内膜炎，糖皮质激素仅能缓解症状，尽早采用针对白血病的化疗才能使患者真正获益[4]–[6]。16号染色体和21号染色体易位形成TLS::ERG融合基因，在AML中仅占1％，预后差，形态学表现为嗜酸性粒细胞增多[60]。本研究中例6经骨髓MICM检查确诊为TLS::ERG（+）AML，外周血极度增高的嗜酸性粒细胞侵袭心内膜引起血栓形成及舒张性心力衰竭，糖皮质激素及羟基脲治疗缓解症状，随后IAG方案化疗使得疾病缓解，并顺利接受allo-HSCT，遗憾的是患者最终因AML复发合并感染死亡。

HES相关的器官损伤几乎可以出现在所有系统，皮肤受累最为常见，而超过半数的患者存在多个器官受累，包括心血管及神经系统[61]。Mayo的研究发现46％的HES患者因心脏受累或血栓栓塞死亡[62]，因此对于疾病的早期识别以及多学科协作尤为重要。2023–2024年文献报道的49例Löeffler心内膜炎病例，有11例合并心脏以外的其他器官浸润，包括5例脑梗死。本研究中例2因言语不清初诊于神经内科，诊疗过程中发现嗜酸性粒细胞增多及心脏受累，经血液科会诊排除克隆性HES，糖皮质激素、抗凝治疗后好转；例3并无头晕失语等神经系统症状，在对HES可能受累器官的评估过程中发现脑梗死。因此在出现嗜酸性粒细胞增多时，不仅要积极寻找病因，多器官系统评估亦非常必要。

综上，以Löeffler心内膜炎为主要临床特征的嗜酸性粒细胞增多症较为罕见，临床表现无特异性，超声心动图或CMR检查具有诊断价值，多个器官系统浸润评估及多学科协作尤为重要。此外，Löeffler心内膜炎病因复杂，特发性因素最为常见，亦有部分为血液肿瘤所致，条件允许时应完善骨髓MICM检查有助于病因诊断。
